# Serology for visceral leishmaniasis: How trusty is the accuracy reported by the manufacturers?

**DOI:** 10.1590/0037-8682-0358-2022

**Published:** 2023-02-20

**Authors:** Mariana Lourenço Freire, Maria Clara de Oliveira Gonçalves, Allana Carolina Marques da Silva, Gláucia Cota, Ana Rabello, Tália Santana Machado de Assis

**Affiliations:** 1 Fundação Oswaldo Cruz, Instituto René Rachou, Pesquisa Clínica e Políticas Públicas em Doenças Infecciosas e Parasitárias, Belo Horizonte, MG, Brasil.; 2 Centro Federal de Educação Tecnológica de Minas Gerais, Contagem, MG, Brasil.

**Keywords:** Visceral leishmaniasis, Diagnostic tests, Performance, Accuracy, Validation

## Abstract

Timely and accurate diagnosis is one of the strategies for managing visceral leishmaniasis (VL). Given the specificities of this infection, which affects different vulnerable populations, the local assessment of the accuracy of the available diagnostic test is a requirement for the good use of resources. In Brazil, performance data are required for test registration with the National Regulatory Agency (ANVISA), but there are no minimum requirements established for performance evaluation. Here, we compared the accuracy reported in the manufacturer’s instructions of commercially available VL-diagnostic tests in Brazil, and the accuracies reported in the scientific literature which were obtained after test commercialization. The tests were identified via the electronic database of ANVISA, and their accuracy was obtained from the manufacturer’s instructions. A literature search for test accuracy was performed using two databases. A total of 28 VL diagnostic tests were identified through the ANVISA database. However, only 13 presented performance data in the manufacturer’s instructions, with five immunoenzymatic tests, three indirect immunofluorescence tests, one chemiluminescence test, and four rapid tests. For most tests, the manufacturers did not provide the relevant information, such as sample size, reference standards, and study site. The literature review identified accuracy data for only 61.5% of diagnostic tests registered in Brazil. These observations confirmed that there are significant flaws in the process of registering health technologies and highlighted one of the reasons for the insufficient control of policies, namely, the use of potentially inaccurate and inappropriate diagnostic tools for a given scenario.

## INTRODUCTION

Visceral leishmaniasis (VL) is a neglected tropical disease caused by *Leishmania*, a protozoan parasite. The disease is considered a worldwide public health problem, with 12 to 65 thousand new cases reported each year between 1998 and 2021 in 80 endemic countries^1^. In Brazil, 38,634 cases of VL were reported in the last 10 years, with an annual average of 277 deaths each^2^. The infection has a high fatality rate (estimated at approximately 6% annually)^2^ and is often related to several socioeconomic indicators. Moreover, restricted access to early and accurate diagnosis and difficulties in clinical suspicions has added to the problem^3^. To manage VL and increase the efficiency of disease control, the availability of diagnostic tests that are simple to perform, accessible, inexpensive, sensitive, and specific is crucial^4^.

Parasitological confirmation remains the reference standard test for VL. However, the invasive nature of the procedure for obtaining a clinical specimen, the need for a specialized health professional, and only moderate sensitivity of the test are limiting factors. Serological tests, with their advantages of high accuracy, simplicity to use, and low cost, are now recognized as a cost-effective strategy for VL diagnosis, at least among patients who are not immunosuppressed. However, significant variations in test accuracy depending on the endemic region, the antigen used, and the age and immune status of the patient must be considered^5^ ^,^ ^6^. Therefore, studies that evaluate the accuracy of these tests in different clinical scenarios are essential before the new health technology can be implemented in clinical practice^7^ ^,^ ^8^.

Ideally, the routine use and development of a new diagnostic test should be supported by a sequence of properly planned studies. A proof-of-concept study should be followed by analytical and precision parameter assessments, followed by clinical performance validation in real-life scenarios, which must include patients with the suspected target condition and submitted to the index and the reference standard tests in parallel in a blinded design (Leeflang et al., 2019).

In Brazil, the registration of diagnostic tests is regulated by The National Health Surveillance Agency (Agência Nacional de Vigilância Sanitária, ANVISA). This requires an accuracy evaluation study before the commercialization of the test. However, the minimum parameters for validation studies are not established which leads to the commercialization of tests without a proper accuracy assessment^9^. Indeed, for VL, a potentially fatal disease if untreated, the use of a rapid diagnosis strategy is crucial. As such, a guideline with the necessary requirements for conducting validation studies to be adopted by the manufacturers must be developed. With concerns about the inaccuracy of the information provided by the manufacturers and, ultimately, of the commercially available tests themselves, we compared the accuracy of commercial VL tests reported by the manufacturers with the tests reported in the scientific literature.

## METHODS

VL diagnostic tests registered in Brazil were identified using the electronic platform of the Brazilian Health Regulatory Agency, ANVISA (https://www.gov.br/anvisa/pt-br), which provides free access to the registered product database. The search ended in June 2021 and was oriented towards registered diagnostic products for VL (identified by the following keywords: Leish, *Leishmania*, Leishmaniasis, Kala-azar, Kalazar, and visceral leishmaniasis). Once the registered tests were identified, information about accuracy was obtained from the manufacturer’s instructions available on the ANVISA website or requested directly from the manufacturer/distributor.

The accuracies of the registered tests were also recovered from the scientific literature on the American Health Library, Medline database (accessed via PubMed, https://www.ncbi.nlm.nih.gov/), and Google Scholar indexer (accessed via https://scholar.google.com.br/). The search strategy was based on the commercial names of the registered tests. Initially, the titles and abstracts of all recovered articles, up to July 2021, were independently read by two researchers. Studies reporting the sensitivity and/or specificity values of any of the registered tests were included. Studies using only non-human samples and/or those published in languages other than English, Portuguese, or Spanish were excluded. Papers were selected for full-text reading based on the inclusion and exclusion criteria. All discrepancies were resolved by consensus after discussion between the two researchers or by consideration of a third researcher if necessary. In this step, duplicates were removed manually. The full read of the selected studies was performed by the same two researchers to confirm their eligibility and extract data, or to exclude if exclusion criteria were identified at that time. All references cited in the included articles were assessed to identify other potential articles. In addition to the sensitivity and specificity values, information such as the type of biological sample used to perform the test (blood or serum), the sample size, the reference standard test, and the country where the study was conducted were extracted from scientific articles and from the manufacturer’s instructions.

The data were compiled in Microsoft Excel spreadsheets, and statistical analyses were performed using MedCalc Statistical Software (MedCalc Software Ltd., Ostend, Belgium)^10^. Accuracy, expressed by sensibility and specificity, presented in the instruction manual of each test, was compared to the performance reported in the literature using a comparison of proportions (chi-squared test). Statistical significance was set at p < 0.05 significance level^11^ ^,^ ^12^. 

## RESULTS

A total of 28 records referring to 26 tests registered for VL were identified in the ANVISA database: nine rapid diagnostic tests (RDT), five indirect immunofluorescent reactions (IIF), nine immunoenzymatic tests (ELISA), one chemiluminescence test (CH) and two tests with unidentified methodologies (Table 1). Ten out of the total (38.5%) were manufactured in Brazil, and the other 16 were produced in Germany (5), Spain (4), the USA (3), Australia (2), China (1), and France (1).

**TABLE 1:** Tests registered for visceral leishmaniasis identified in the Brazilian health regulatory agency.

Product	Type	Distributor	Manufacturer	Country of origin	Record number
IT LEISH	Rapid test	Diamed Latino América S.A.	BIO-RAD	France	80004040138
Kalazar Detect™	Rapid test	Fundação de Apoio ao Hospital Universitário Cassiano Antonio Moraes	Inbios International	USA	80123410002
*Leishmania* Ab Rapid Test	Rapid test	Diagnóstica Indústria e Comércio	Diagnóstica Indústria e Comércio	Brazil	80638720087
Leishmaniose LF	Rapid test	Advagen Biotech LTDA	Advagen Biotech LTDA	Brazil	81472060018
Leishmaniose VH BIO	Rapid test	QuibasaQuímicaBásica LTDA	QuibasaQuímicaBásica LTDA	Brazil	10269360334
Leishmaniose Visceral Rápido	Rapid test	Vida Biotecnologia LTDA ME	Vida Biotecnologia Ltda ME	Brazil	80785070047
Teste Rápido Leishmaniose Bahiafarma	Rapid test	Fundação Baiana de Pesq. Científica e Desenv. Tecnológico, Fornecimento e Distribuição de Medicamentos - Bahiafarma	Fundação Baiana de Pesq. Científica e Desenv. Tecnológico, Fornecimento e Distribuição de Medicamentos - Bahiafarma	Brazil	81285200008
OnSite Leishmania IgG/IgM Combo Rapid Test	Rapid test	BIO Advance Diagnosticos LTDA	BEIJING GENESEE BIOTECH	China	80524900058
OL Leishmaniose Visceral Humana	Rapid test	Chembio Diagnostic Brazil LTDA	Chembio Diagnostics Brazil LTDA.	Brazil	80535240013
IF: *Leishmania donovani* IgG	IIF	Euroimmun Brasil Medicina Diagnostica	Euroimmun AG	Germany	81148560064
IF: *Leishmania donovani* IgM	IIF	Euroimmun Brasil Medicina Diagnostica	Euroimmun AG	Germany	81148560065
IFI Leishmaniose Humana	IIF	Fundação Oswaldo Cruz - Biomanguinhos	Fundação Oswaldo Cruz	Brazil	10106330011
*Leishmania* IFA IgG	IIF	Medivax Indústria e Comércio LTDA	IVD Research	USA	10259610062
*Leishmania* IFA IgG	IIF	Resserv Comércio de Produtos Diagnósticos	Vircell	Spain	80213250222
		VirionDiagnostica LTDA			80263710018
Biolisa Leishmaniose Visceral	ELISA	Quibasa Química Básica	QuibasaQuímicaBásica	Brazil	10269360317
*Leishmania* CEL	ELISA	RCS Comércio de Produtos em Diagnóstico LTDA	Cellabs PTY	Australia	80009070041
*Leishmania* ELISA IgG + IgM	ELISA	Resserv Comércio de Produtos Diagnósticos	Vircell	Spain	80213250196
		VirionDiagnostica LTDA			80263710004
*Leishmania* VISCERAL IgG CELISA	ELISA	RCS Comércio de Produtos em Diagnóstico LTDA	Cellabs PTY	Australia	80009070044
Leishmaniose MAX IgG	ELISA	Advagen Biotech LTDA	Advagen Biotech LTDA	Brazil	81472060019
Novalisa TM *Leishmania infantum* IgG - ELISA	ELISA	Argoslab Distribuidora de Produtos para Laboratórios LTDA	Novatec Immundiagnostica	Germany	80464810379
RIDASCREEN® *Leishmania* Ab	ELISA	Resserv Comércio de Produtos Diagnósticos	R-BIOPHARM	Germany	80213250451
SERION ELISA Classic *Leishmania* IgG	ELISA	Serion Brasil Importação e Distribuição de Produtos Diagnósticos LTDA	Institut Virion\Serion	Germany	80826840079
Teste para determinação de anticorpos IgG para *Leishmania infantum* LIG153	ELISA	B.T.I Biotecnologia Industrial LTDA	B.T.I Biotecnologia Industrial LTDA	Brazil	80049570027
*Leishmania* Virclia IgG + IgM Monotest	CH	VirionDiagnostica LTDA	Vircell	Spain	80263710051
KIT Qualicode Chagas/*Leishmania*	N/A	D-MED Material Médico Laboratorial LTDA	Immunetics INC	USA	10327810022
Melotest *Leishmania* Ab	N/A	Laboratorio PAS Comercial LTDA	Melotec S.A.	Spain	10287380071

**Legend: IFA:** Indirect immunofluorescence reaction; **ELISA:** Enzyme-linked immunosorbent assay; **CH:** Chemiluminescence; **N/A:** not available.

**FIGURE 1:**
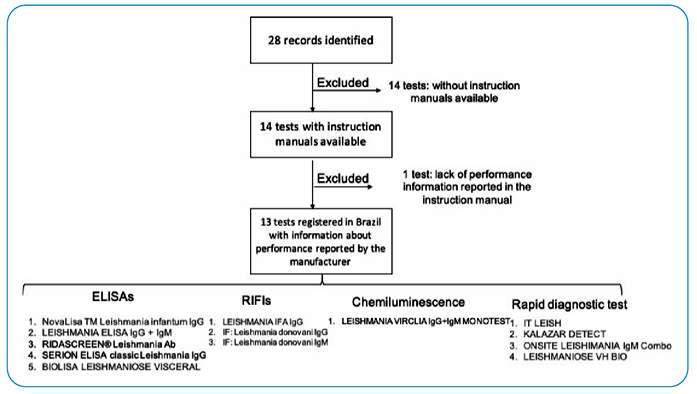
The tests for visceral leishmaniasis included in this study.

For the 14 tests, an instruction manual was obtained: only one was available on the ANVISA website, and the others were recovered through direct contact with the manufacturer/distributor. After individual analysis of the obtained instruction manuals, the IFI Human Leishmaniasis test (Fundação Oswaldo Cruz - Biomanguinhos) was excluded because of the absence of data regarding the performance study in the instruction manual. Therefore, a total of 13 VL diagnosis tests fulfilled the criteria and were included, with five ELISAs, three IIF, one chemiluminescence test, and four RDT (Figure 1).

Regarding the 13 diagnostic tests included in this review, only eight (61.5%) had validation analyses available in the scientific literature. RDTs, especially IT LEISH and Kalazar Detect (Table 2), were the most evaluated in validation studies worldwide. For the IT LEISH, the validation study reported in the manufacturer's instruction manual was performed in India, and 99% and 100% sensitivity and specificity were reported, respectively. Scientific studies carried out in this same region also reported a sensitivity of 96.2 to 100%. On the other hand, in African countries, such as Sudan, Ethiopia, and Kenya, lower sensitivity rates have been reported, between 83.8 and 96.8%. In Brazil, the reported sensitivity rates ranged from 93.3 to 100%. The accuracy of IT LEISH seems to be independent of the biological sample used, if serum or blood samples were used. For Kalazar Detect, a sensitivity rate of 89.8% was observed in a validation study performed in Brazil. No statistical differences were detected in relation to other validation studies carried out in American regions, with sensitivities ranging from 85.5 to 90%, except for the study conducted by Moura et al. (2013)^13^ in Brazil, in which the sensitivity was 72.4%. However, it is important to note that in this specific study, several reference tests were used, such as direct test or culture and/or IFA and/or test therapy (presumption of diagnosis based on the response after the instruction of specific therapy). Regarding Onsite Leishmania IgG/IgM Combo, only four studies performed in different countries were retrieved. For this test, two performances are informed in the manufacturer’s instructions based on two different reference test criteria: IgG or IgM positivity in another serology. 

**TABLE 2:** Rapid test performance reported in the manufacturer’s instructions and in scientific literature.

Reference - sample	Sensitivity % (CI 95%)	p-value	Specificity (CI 95%)	p-value	Number of cases	Number of non-cases	Reference standard	Country
IT LEISH								
Instruction manual	99	-	100	-	206	269	NA	India
Ritmeijer et al.^14^ - blood	89.6 (84.5-93.4)	<0.00*	99.2 (95.9-100)	0.14	201	133	Parasitological	Sudan
Sundar et al.^15^ - blood	99 (94-100)	1.00	100	1.00	100	54	Parasitological	India
Sundar et al.^15^ - serum	100 (95-100)	0.32	95	0.00*	100	150	Parasitological	India
Machado de Assis et al.^16^ - blood	93 (89.2-96.4)	0.00*	97 (91.6-99)	0.00*	213	119	Parasitological	Brazil
Mandal et al.^17^ - serum	100	0.69	87	<0.00*	16	40	Parasitological	India
Neto et al.^18^ - serum	100 (85-100)	0.58	100 (85-100)	1.00	30	60	Parasitological	Brazil
Abass et al.^19^ - serum	83.8 (76.7-90.8)	<0.00*	93.0 (89.3-96.6)	<0.00*	115	177	Parasitological or clinical + DAT + therapeutic test	Sudan
Machado de Assis et al.^20^ - blood	93.0 (89.2-96.4)	0.00*	97 (92.0-99.1)	0.00*	213	119	Parasitological	Brazil
Cañavate et al.^21^ - blood	91.4 (80.7-100)	0.00*	94.0 (87.6-100)	0.00*	36	66	Parasitological or PCR	Ethiopia
Cunningham et al.^5^ - serum	92.0 (87.8-94.8)	0.00*	95.6 (92.2-97.5)	0.00*	250	250	Parasitological	Brazil, Africa and India
Peruhype-Magalhães et al.^22^ - serum	93.3 (89.0-96.4)	0.00*	96.5*(90.0-99.3)	0.00	197	83	Parasitological	Brazil
Machado de Assis et al.^23^ - serum	94.0 (90.1-96.3)	0.00*	100 (97.0-100)	-	404	-	Analysis of latent classes	Brazil
Mbui et al.^24^ - serum	89.3 (82.7-94.0)	0.00*	89.8 (81.5-95.2)	0.00*	131	88	Parasitological	Kenia
Kumar et al.^25^ - blood	98.7 (95.3-99.8)	0.79	99.2 (95.4-100)	0.14	150	119	Parasitological	India
Kumar et al.^25^ - serum	98.7 (95.3-99.8)	0.79	99.2 (95.4-100)	0.14	150	119	Parasitological	India
Kumar et al.^25^ - blood	100 (87.2-100)	0.60	93.4 (88.2-96.8)	<0.00*	27	152	Parasitological	Nepal
Kumar et al.^25^ - serum	96.3 (81.0-99.9)	0.25	92.8 (87.4-96.3)	<0.00*	27	152	Parasitological	Nepal
Bezuneh et al.^26^ - serum	96.8 (91.1-99.3)	0.18	98.2 (93.6-99.8)	0.03*	82	111	Parasitological	Ethiopia
Abbas et al.^27^ -serum	88.0 Sudan	---	97.5 Sudan	---	142	89	Parasitological	Sudan, India and France
	96.2 India	---	96.6 India	---				
	88.5 France	---	---	---				
Freire et al.^8^ - serum	96.3 (89.6-98.7)	0.12	96.2 (89.4-98.7)	0.00*	80	79	Parasitological	Brazil
Kassa et al.^28^ - serum	95.0 (83.5-98.6)	0.07	100 (91.2-100)	1.00	40	40	Parasitological and PCR	Ethiopia
Sanchez et al.^6^ - serum	94.4 (88.8-97.2)	0.01*	97.2* (92.0-99.0)	0.01	124	106	Parasitological or DAT	Brazil
Lévêque et al.^29^ - serum	85.1 (81.2-88.9)	0.00*	99.3 (98.3-100.2)	0.17	202	138	Parasitological and PCR	France, Tunisia and Morocco
**Kalazar Detect**								
**Manufacturer’s instruction - serum**	**89.8 (82.9-94.3)**	-	**100 (92.3**-**100)**	-	**128**	**59**	**Parasitological**	**Brazil**
Schallig et al.^30^ - serum	85.5	0.56	82.0	0.00*	21	19	Parasitological	Brazil
Carvalho et al.^31^ - serum	90.0	0.96	100	1.00	128	60	Parasitological	Brazil
Boelaert et al.^32^ - serum	87.4 (81.7-91.9)	0.52	77.0 (68.6-84.0)	0.01*	181	126	Parasitological	Nepal
Chappuis et al.^33^ - serum	82.0 (74.0-87.0)	0.07	99.0 (95.0-100)	0.44	131	112	Parasitological or DAT	Uganda
Sundar et al.^15^ - serum	99.0 (95.0-100)	0.00*	89.0 (86.0-920)	0.01*	150	358	Parasitological	India
Alborzi et al.^34^ - blood or serum	82.4	0.19	100	1.00	47	161	Parasitological	Shiraz
Sundar et al.^15^ - serum	98.0 (93-100)	0.01*	97.0	0.18	100	150	Parasitological	India
Diro et al.^35^ - serum	71.7 (56.3-83.5)	0.00*	82.4 (68.6-91.1)	0.00*	49	52	Parasitological	Ethiopia
Takagi et al.^36^ - serum	93.2	0.42	---	---	74	Not provided	Parasitological	Bangladesh
Boelaert et al.^37^ - serum	75.4 (55.9-90.5) Ethiopia	---	70.0 (46.3-88.9) Ethiopia	---	38		Analysis of latent classes	Ethiopia, Kenya, Sudan, India and Nepal
	84.7 (78.6-89.8) Kenya	---	89.9 (83.2-95.1) Kenya	---	308			
	77.9 (69.2-85.6) Sudan	---	91.8 (86.7-96.2) Sudan	---	294			
	99.6 (98.4-100) India	---	90.0 (81.2-96.4) India	---	352			
	96.5 (92.1-99.2) Nepal	---	90.9 (80.8-97.5) Nepal	---	158			
Welch et al.^38^ - serum	90	0.96	100	1.00	94	78	IFA	United States
Ozerdem et al.^39^ - serum	69.2	0.05	97.2	0.20	10	40	Parasitological	Turkey
Saghrouni et al.^40^ - serum	87.1	0.40	94.4	0.06	574	355	Parasitological	Tunisia
Pattabhi et al.^41^ - serum	88.7 (76.97-95.73)	0.82	100 (91.19-100)	1.00	62	75	Parasitological	Sudan
Teran-Angel et al.^42^ - serum	94.2 (87.7-100)	0.36	100	1.00	50	42	ELISA	Venezuela
Cañavate et al.^21^ - blood	94.3 (85.2-100)	0.42	98.5 (94.9-100)	0.35	34	68	Parasitological or PCR	Ethiopia
Chakravarty et al.^43^ - urine	96.4 (94-99)	0.00*	66.7 (55-78) Healthy individuals from an endemic area	<0.00*	280	48	Parasitological	India
			77.08 (65-89) Healthy individuals from a nonendemic area	0.00*		66		
			62.2* (48-76) No cases with other diseases	<0.00*		45		
Chakravarty et al.^43^ - serum	100 (98-100)	0.00*	92.4 (83.4-96.7) Healthy individuals from endemic areas	0.03*	280	48		
			100 (98-100) Healthy individuals from nonendemic areas	1.00		66		
			95.55 (85.2-98.8) No cases with other diseases	0.10		45		
Singh et al.^44^	98 (93-100)	0.00*	89(82-97)	0.01*	150	305	Parasitological	India
El Moamly et al.^45^ - serum	89 (78-99)	0.89	92(85-99)	0.03	35	63	Parasitological	Saudi Arabia
			91.5 (80.1-96.6) Healthy individuals from non-endemic areas	---				
Vaish et al.^46^ - saliva	82.5 (74.5-88.3)	0.10	91.6 (84.3-97.7) Healthy individuals from endemic areas	---	114	186	Parasitological	India
			80.1 (70.6-92.1) No cases with other diseases	---				
			100 (92.4-100) Healthy individuals from non-endemic areas	1.00		47		
Vaish et al.^46^ - serum	100 (96.7-100)	0.00*	94.7 (88.3-97.7) Healthy individuals from endemic areas	0.07	114	95	Parasitological	India
			95.5 (84.9-98.7) No cases with other diseases	0.10		44		
Singh et al.^47^-urine	96.1 (93.6-97.8)	0.01*	100 (97-100)	1.00	365	421		
			93.8 (88.9-97.0) No cases of endemic areas	0.05		162		
Singh et al.^47^ -serum	100 (98.9-100)	0.00*	100 (97.6-100) No cases of non-endemic areas	1.00	365	154	Parasitological	India
			96.2 (90.5-98.9) No cases with other diseases	0.13		105		
Cunningham et al.^5^ - serum	84.7 (79.7-88.7)	0.17	96.8 (93.9-98.4)	0.16	250	250	Parasitological	Brazil, Africa and India
Peruhype-Magalhães et al.^22^ - serum	88.1 (83-92.3)	0.64	90.6 (82.3-96.0)	0.02*	197	83	Parasitological	Brazil
Kumar et al.^25^ - blood	99.3 (96.3-100)	0.00*	97.5 (92.8-99.5)	0.22	150	119	Parasitological	India
Kumar et al.^25^ - serum	99.3 (96.3-100)	0.00*	99.2 (95.4-100)	0.49	150	119	Parasitological	India
Kumar et al.^25^ - blood	96.3 (81.0-99.9)	0.29	94.1 (89.1-97.3)	0.06	27	152	Parasitological	Nepal
Kumar et al.^25^ - serum	96.3 (81.0-99.9)	0.29	94.1 (89.1-97.3)	0.06	27	152	Parasitological	Nepal
Moura et al.^13^ - serum	72.4 (64.6-79.0)	0.00*	99.6 (97.6-99.9)	0.63	145	236	Parasitological or culture and/or IFA and/or therapeutic test	Brazil
Bezuneh et al.^26^ - serum	92.6 (85.4-96.9)	0.49	98.2 (93.6-99.8)	0.30	82	111	Parasitological	Ethiopia
Ghosh et al.^48^ - blood	100	0.07	96.92 (89.30-99.54)	0.17	30	65	Parasitological or clinical	Bangladesh
Bangert et al.^49^ - serum	78.0 (70.8-85.2)	0.00*	100 (99.8-100)	1.00	405	338	Parasitological	Spain
Herrera et al.^50^ - serum	91.5 (83.4-95.8)	0.68	89.2(80.1-94.4)	0.01*	82	74	IFA	Colombia
Freire et al.^8^ - serum	92.5 (84.6-96.5)	0.51	94.9 (87.7-98.0)	0.08	80	79	Parasitological	Brazil
Kassa et al.^28^ - serum	95.0 (83.5-98.6)	0.32	92.5(80.1-97.4)	0.04*	40	40	Parasitological and PCR	Ethiopia
Sanchez et al.^6^ - serum	87.9 (81.0-92.5)	0.63	93.4(87.0-96.8)	0.04*	124	106	Parasitological and DAT	Brazil
**OnSite Leishmania IgG/IgM Combo Rapid Test**								
Manufacturer’s instruction^#^ - IgM	**91.2**	---	**99.5**	---	**34**	**200**	***L. donovani* IgM - EIA commercial**	**Not provided**
**Manufacturer’s instruction - IgG**	**92.9**	---	**99.0**	---	**14**	**200**	***L. donovani* IgG - EIA commercial**	**Not provided**
Cunningham et al.^5^	99.6 (97.8-99.9)	<0.00*	96.8 (93.8-98.4)	0.04*	250	249	Parasitological	India
Freire et al.^7^	91.2 (84.5-95.1)	1.00	94.5 (86.7-97.9)	0.00*	186	186	Parasitological	Brazil
Kassa et al.^28^	100 (91.2-100)	0.05	77.5 (62.5-87.7)	<0.00*	40	40	Parasitological and PCR	Ethiopia
Ortalli et al.^51^	63.0 (42.0-80.0)	0.00*	98.0 (88.0-100)	0.29	27	50	PCR	Italy
**Leishmaniose VH BIO**								
**Manufacturer’s instruction** **serum**	**99.1 (97.3-100)**	---	**>99.9 (91.0-100)**	---	**110**	**100**	**Not provided**	**Not provided**

* p < 0.05. # The IgM accuracy reported in the manufacturer instruction was used to compare with the literature studies, since this antibody is often interpreted as an indicator of acute infection.

Among the four IIF tests included in this study, only one study assessed the accuracy of Leishmania IFA IgG (Table 3). Overall, the manufacturer’s instructions lacked relevant information regarding how validation studies were conducted. In some cases, as observed for *Leishmania* IFA IgG and *Leishmania* VIRCLIA IgG+IgM MONOTEST, data about the population, such as the sample size and the country from which the samples come, are missing. For other tests, such as IF *Leishmania donovani* IgG and IgM, there is no information about the reference standard tests. Similarly, some ELISAs, such as NovaLisa *Leishmania infantum* IgG and RIDASCREEN *Leishmania* Ab, lack information about the reference standard and population included (Table 4). For the other ELISAs, *Leishmania* ELISA IgG + IgM, SERION ELISA classic *Leishmania* IgG, and Biolisa Leishmaniose Visceral, there was no information about the country where the manufacturing study was conducted. These limitations hamper critical evaluation by comparing the manufacturer and literature accuracy.

**TABLE 3:** Performance of immunofluorescence and chemiluminescence reaction tests reported in manufacturer’s instructions and scientific studies.

Reference	Sensitivity	P - value	Specificity	P - value	Number of cases	Number of no cases (controls)	Reference standard	Country	Type of test
***Leishmania* IFA IgG**									
**Manufacturer’s instruction - serum**	**100**	---	**100**	---	**NA**	**NA**	**IFA**	**NA**	**IFA**
Freire et al.^8^	78.8 (68.6-86.3)	---	96.2 (89.4-98.7)	---	80	79	Parasitological	Brazil	
**I F: *Leishmania donovani* IgG**									
**Manufacturer’s instruction - serum**	**100**	---	**99**	---	**10**	**200**	**NA**	**Germany**	**IFA**
**IF: *Leishmania donovani* IgM**				
**Manufacturer’s instruction - serum**	**80**	---	**97.5**	---	**10**	**200**	**NA**	**Germany**	**IFA**
***Leishmania* VIRCLIA IgG+IgM MONOTEST**									
**Manufacturer’s instruction - serum**	**92**	---	**99**	---	**NA**	**NA**	**ELISA**	**NA**	**Chemo**

**TABLE 4:** Performance values of immunoenzymatic assays reported in manufacturer’s instructions and literature.

Reference	Sensitivity	P - value	Specificity	P - value	Number of cases	Number of non cases	Reference standard	Country
**NovaLisa *Leishmania infantum* IgG - ELISA**								
**Manufacturer’s instruction**	**91**	---	**85**	---	**NA**	**NA**	**NA**	**NA**
Freire et al.^8^	86.3 (77.0-92.2)	---	96.2 (89.4-98.7)	---	80	79	Parasitological	Brazil
Stensvold et al.^52^	95.5 (77.2-99.9)	---	81.0 (58.1-94.6)	---	43	43	PCR	Denmark
Lévêque et al.^29^	89.5 (86.1-92.9)	---	96.4 (94.3-98.4)	---	202	138	Parasitological and PCR	France, Tunisia and Morocco
***Leishmania* ELISA IgG + IgM**								
**Manufacturer’s instruction**	**97 (83-99)**	---	**99 (95-100)**	---	**138 total**	**138 total**	**IFA**	**NA**
Kiliç et al.^53^	95.8	0.76	82.9	<0.00*	24	35	Parasitological	Turkey
Mandal et al.^17^	100	0.48	87	0.00*	16	40	Parasitological	India
Mniouil et al.^54^	75	<0.00*	95.8	0.23	24	25	Parasitological	Morocco
Freire et al.^8^	77.5 (67.2-85.3)	<0.00*	93.7 (86.0-97.3)	0.03*	80	79	Parasitological	Brazil
Ortalli et al.^51^	74.0 (53.0-88.0)	<0.00*	98.0 (88.0-100)	0.59	27	50	PCR	Italy
**RIDASCREEN *Leishmania* Ab**								
**Manufacturer’s instruction**	**100**	---	**100**	---	**NA**	**NA**	**NA**	**NA**
Harizanov et al^.^ ^55^	98.3	---	- -	---	59	---	Parasitological	Bulgaria
Freire et al.^8^	93.8 (86.2-97.3)	---	77.2 (66.8-85.1)	---	80	79	Parasitological	Brazil
Lévêque et al.^29^	80.7 (76.3-85.0)	---	99.3 (98.3-100.2)	---	202	138	Parasitological and PCR	France, Tunisia and Morocco
**SERION ELISA classic *Leishmania* IgG**								
**Manufacturer’s instruction**	**>99**	---	**>99**	---	**203 total**	**203 total**	**ELISA**	**NA**
Kassa et al.^28^	100.0 (91.2-100)	0.53	97.5 (87.1-99.6)	0.44	40	40	Parasitological and PCR	Ethiopia
**Biolisa Leishmaniose Visceral**								
**Manufacturer’s instruction**	**97.9**	---	**99**	---	**49**	**51**	**ELISA**	**NA**

* p < 0.05.

## DISCUSSION

The accuracy of serological tests for VL is determined by factors related to the patients, such as their immune status and age, disease severity, and other factors, such as the *Leishmania* species involved, the test technique, and antigens used as targets. In addition, the adopted reference standard test^5^ ^,^ ^8^ ^,^ ^37^ and other methodological aspects of the validation study may also influence the accuracy estimation. There are many requirements for producing reliable estimates of test accuracy. Indeed, the process of validation and registration with regulatory agencies must be carefully evaluated. Comparisons between the accuracy reported by the manufacturer and those observed in clinical studies are essential to confirm the diagnostic accuracy under real conditions in the field, identify technologies with accuracies lower than expected prior to incorporation in clinical practice, and reduce diagnostic inaccuracy and public health risks.

In Brazil, manufacturers must follow a specific resolution before submitting a registration request to ANVISA. Among the requirements are the presentations of the analytical and clinical accuracy data, included in a technical dossier and in the test leaflet^9^. These studies should provide accurate information such as sensitivity, specificity, accuracy, and diagnostic precision. However, the minimum criteria defining methodological requirements, such as sample size, sample characterization, and reference test, have not been established, allowing the registration of poorly evaluated tests. In addition, several methodological information regarding the validation study were missing, such as the reference standard, and the number of included and excluded cases were not included in the manufacturer’s instructions, hampering the correct interpretation of the results. Overall, the sensitivity and specificity rates reported by the manufacturer were obtained from analytical validation studies based on uncalculated samples composed of selected cases and controls, which do not represent the clinical diversity (clinical spectrum) of real scenarios, tending to overestimate performance. 

The validation of a test should qualify for use in clinical decision-making. After analytical validation, true characterization of the performance of the test regarding its intended use (clinical validation) should be carried out following the Standards for Reporting Diagnostic Accuracy Studies (STARD)^56^. Analytical validity is the test’s ability to measure the status of a sample accurately and reliably in the laboratory, and it includes three different phases of test development: pre-analytical, analytical, and post-analytical phase^57^. Clinical validation should demonstrate how robust and reliable the test results correlate with the clinical outcomes of interest. In addition to clinical validity, which implies the appropriate distinction of cases and not cases, new perspectives have been raised as equally important in evaluating the usefulness of a test: the concept of the fit-for-purpose. This concept ensures that the test performs robustly according to predefined epidemiological and clinical parameters and facilitates the establishment of definitive acceptance criteria for clinical use (validation of clinical utility)^58^. 

The difference in accuracy among regions has been widely verified for VL, generally associated with the diversity of parasite species and/or title antibodies, which has been related to different genetic factors, age patterns, immune response, and nutritional status of patients^5^ ^,^ ^32^. Mainly for IT LEISH and Kalazar Detect, the highest rates of sensitivity and specificity were observed for studies conducted in India when compared to other endemic regions, like Brazil and East Africa. This finding confirms the importance and necessity of local validation studies prior to the commercially available VL-test, preventing them from being used in clinical decision-making.

It is important to highlight the limitations of studies evaluating IFIs registered in Brazil, especially considering that this technique has been available and recommended for VL diagnosis for a long time by the Brazilian Visceral Leishmaniasis Surveillance and Control Program of the Ministry of Health (MS)^59^. Although some studies describing the accuracy of this IFI are available^8^ ^,^ ^22^, a comparative analysis of the sensitivity and specificity rates described by the manufacturer was not possible because of the unavailability of these parameters in the manufacturer's instructions. ELISAs are generally used in private laboratories in Brazil, with few local validation studies corroborating their use. 

Regardless of region, estimates of sensitivity and specificity may often vary between studies due to differences in the study population as a result of demographic or other covariate factors, such as disease stage and the presence of comorbidities. Thus, there were two main sources of bias related to the population evaluated: selection and confounding bias^61^. More importantly, the diagnostic test performance may vary with the prevalence of the disease in the evaluated population. Based on mathematical definitions, sensitivity and specificity do not depend on disease prevalence; however, this is an outdated paradigm^60^. The influence of prevalence can occur due to intervenient features, such as patient spectrum, referral filter, reader expectation, and artifactual mechanisms, which include distorted inclusion of participants, verification bias, and reference standard misclassification or misuse. 

In fact, the selection of reference standards is a crucial but challenging element that influences test performance. Generally, the gold standard test is nonexistent, and consequently, the sensitivity and specificity rates can be over-or underestimated according to the frequency of misclassifications made by the reference standard and the degree of correlation of errors between the index test and reference standard^61^. For VL, a parasitological test is generally used because of its high specificity. However, the variable and usually lower sensitivity can affect the accuracy of the index test. The use of an index and reference test of the same methodology, such as immunological methods, presents a tendency to have concordant errors, and in this way, may act by overestimating the accuracy of the evaluated test. To minimize the impact of this error, because a gold standard is not available, it is possible to consider the results of multiple imperfect tests using latent class analysis, as reported by Boelaert et al. (2008)^37^ and Machado de Assis et al. (2012)^23^.

In general, the commercialization of VL diagnostic tests supported by less rigorous validation studies may lead to the availability of poorly performing tests, with serious implications for the diagnosis and prognosis of patients. For VL, this fact causes concerns because false-negative results may delay the treatment of the disease, which may lead to fatality if left untreated. Conversely, false-positive results are also of great concern because of the high toxicity of the available treatments. Importantly, economic losses to the public health systems and to patients may result due to a lack of accuracy^60^ ^,^ ^61^.

The limited information provided by manufacturers regarding the accuracy studies conducted prior commercialization of the tests in Brazil was the major lacuna observed in this review. It is important to highlight that it was not one of our goals to summarize the “correct” sensitivity-specificity of the tests, but rather to verify how different these measures can be. Therefore, we did not perform a systematic review. Instead, we conducted an extensive and careful search using various scientific databases and the reference list of each included article. The results of our data analyses revealed how the accuracy reported by the manufacturers differed from local studies, and how it is necessary to perform a validation study before the use of a VL test in clinical practice. Given the importance of a diagnosis for correct treatment, the establishment of a guideline with minimum criteria for test registration by all regulatory agencies is encouraged. This practice can also be useful for test developers. Indeed, the obligation for local studies with sample calculations supported by the number of participants and the selection of a robust reference standard test may be the preferred way of selecting VL tests with higher accuracy in each endemic area.
